# Comprehensive Review on Viral RNA Extraction Strategies for Enhanced Molecular Diagnostics

**DOI:** 10.1155/ipid/5579320

**Published:** 2025-12-17

**Authors:** I. Made Artika, Chairin Nisa Ma’roef, Edison Johar, Frilasita Aisyah Yudhaputri, Khin Saw Aye Myint

**Affiliations:** ^1^ Department of Biochemistry, Faculty of Mathematics and Natural Sciences, Bogor Agricultural University, Dramaga Campus, Bogor, 16680, Indonesia, ipb.ac.id; ^2^ Exeins Health Initiative, Daerah Khusus Ibukota Jakarta, 12870, Indonesia

**Keywords:** RNA, RNA extraction, RNA isolation, RNA purification, virus

## Abstract

RNA viruses, characterized by their RNA‐based genomes, have garnered significant concern due to their role in numerous lethal pandemics and remain a significant global health threat. Their predominance among emerging pathogens underscores the need for rapid detection and molecular characterization. PCR has been proven to be an indispensable tool for monitoring the emergence and spread of RNA viruses, but its success requires efficient viral RNA isolation. Poor RNA recovery may lead to failed detection, limiting downstream applications such as genome sequencing and molecular epidemiology. This review provides an overview of major RNA extraction strategies, including TRIzol‐based, spin column–based, and magnetic bead–based methods, as well as novel and automated platforms. We compared their advantages, limitations, and trade‐offs, emphasizing compatibility with downstream processes such as PCR and next‐generation sequencing. Challenges related to sample collection, transport, storage, and RNA integrity are discussed, particularly in resource‐limited settings where cold‐chain and biosafety infrastructure may be limited. Additionally, we discussed applications of extracted RNA for diagnostics, viral characterization, and public health interventions, as well as advances in point‐of‐care (POC) technologies that integrate extraction with detection. Future directions focus on developing robust, cost‐effective, and scalable extraction methods that are adaptable to both high‐throughput laboratories and portable POC systems, ensuring global preparedness against emerging RNA viruses.

## 1. Introduction

**Figure 1:**
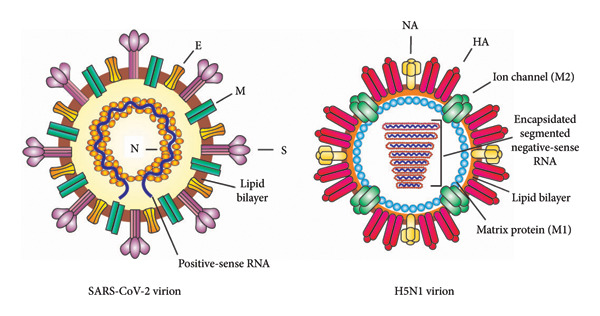
Molecular structure of SARS‐CoV‐2 and influenza A virus, H5N1. SARS‐CoV‐2 is an example of RNA virus with a positive‐sense single‐stranded RNA genome and avian influenza A virus, H5N1, is an RNA virus characterized by a negative‐sense single‐stranded RNA genome. E, envelope protein; M, membrane protein; S, spike protein; N, nucleocapsid phosphoprotein; NA, neuraminidase protein; HA, hemagglutinin protein; M2, ion channel protein; M1, matrix protein.

Virus genome could be made up of either DNA or RNA genetic material and exists in single‐ or double‐stranded forms. A single‐stranded genome is further classified based on the sense or polarity: positive or negative sense [1]. SARS‐CoV‐2 is an example of an RNA virus with a positive‐sense single‐stranded RNA (+ssRNA) genome [2], whereas avian influenza virus (H5N1) is an example of a negative‐sense single‐stranded RNA (–ssRNA) genome [3] (Figure 1). The positive‐stranded viral RNA genome is a functional mRNA which can be directly translated into protein within the host cells. In contrast, the negative strand is an antisense strand that functions as a template for mRNA synthesis [1]. Rotaviruses, the most common cause of life‐threatening gastroenteritis in young children [4], are an example of double‐stranded RNA viruses. In general, replication of RNA viral genomes is carried out by viral replicase complexes involving viral and cellular proteins with RNA‐dependent RNA polymerase (RdRP) as a catalytic subunit [5].

RNA viruses are particularly notorious for their involvement in multiple deadly pandemics and large‐scale epidemics, such as the Spanish flu in 1918 causing more than 50 million deaths [6], the Ebola outbreak from 1976 to 2020 claiming 15,258 lives [6], and the recent coronavirus disease 2019 (COVID‐19) pandemic resulting in over 777 million confirmed cases and over 7 million deaths by January 2025 [7]. Historically, influenza virus A, an RNA virus, is responsible for at least 30 influenza pandemics including the Spanish flu, the Asian flu (1957–1958) with estimated one to two million fatalities, the Hongkong flu (1968–1970) claiming 0.5–2 million deaths, and the swine flu (2009–2010) accountable for 0.5 million fatalities [8]. According to the World Health Organization (WHO), between 2003 and December 2024, as many as 954 cases of confirmed H5N1 influenza resulted in 464 deaths [9]. Recently, cases of human infection with H5N1 were reported to occur in Viet Nam and the USA [10]. Other RNA viruses from the *Flavivirus* genus pose persistent public health threats, such as dengue virus (DENV), West Nile virus (WNV), Zika virus (ZIKV), Japanese encephalitis virus (JEV), tick‐borne encephalitis virus, and Usutu virus. Flaviviruses infect more than 400 million individuals every year [11]. The global incidence of DENV infection has increased exponentially in recent years, with nearly half of the world’s population at risk. Since the ZIKV outbreak in Brazil in early 2015, a total of 89 countries and territories have reported evidence of Zika infection as of December 2021. It is important to note that prenatal ZIKV infection has been linked to birth defects, including microcephaly and other serious brain anomalies. The Zika epidemic was declared a public health emergency of international concern by the WHO in 2016 [12–14].

Despite RNA viruses evolving mechanisms to maintain genome integrity [5], they continue to exhibit high mutation rates to enhance their evolutionary adaptability and virulence [15–17]. The mutation rate of RNA viruses is up to five orders of magnitude higher than that of DNA viruses. Their fast evolutionary rate coupled with short generation time increases the chance of RNA viruses to infect new hosts which may lead to a disease outbreak or even pandemic [18]. The high mutation rate is partly attributed to the low‐fidelity RdRP, with the majority lacking proofreading capacity to prevent nucleotide misincorporation during genome replication [16]. This results in virus populations with high genetic diversity, allowing rapid adaptation to different environmental conditions [17]. Recombination and reassortment phenomena also contribute to the high genetic variability in some RNA viruses [18].

In recent decades, the impact of emerging zoonotic viral diseases, particularly those caused by RNA viruses, has been increasing [19]. Over 224 RNA viruses have been identified to infect humans, with 88% originating from animals [20]. In addition to viral evolution, several factors have been described that contribute to the emergence of novel zoonotic diseases, such as changes in human demographics, human behavior, ecology, and climate [20, 21]. Therefore, a comprehensive understanding of RNA virus infection mechanisms, adaptation, replication, and transmission is crucial for developing better control strategies [18, 22]. Sufficient high‐quality viral genomic RNA is a prerequisite for successful molecular detection and genetic characterization. This review provides an overview of basic principles and the optimization of common techniques employed to isolate and purify viral RNA from human specimens.

## 2. Viral Sample

### 2.1. Sample Type and Collection

One of the critical stages in viral RNA extraction is sample collection. Proper sample collection, transport, and storage are vital to obtain good‐quality samples for successful RNA isolation and subsequent analysis. For diagnostic purposes, the timely collection of appropriate specimens is recommended and should be performed by trained and competent personnel. Safety procedures should be strictly adhered to, and appropriate personal protective equipment should be worn to prevent infection [14, 23]. Samples are typically collected from sites containing the highest concentration of the target virus, usually from the site of infection [24]. The specimen types that can be used to extract RNA from each target virus are shown in Table 1.

**Table 1:** Types of samples suitable for viral RNA extraction.

Target virus	Sample type	References
SARS‐CoV‐2	Nasopharyngeal swab, oropharyngeal swab, bronchoalveolar lavage fluid, sputum, fiberoptic bronchoscope brush biopsy, feces, whole blood, saliva, gargle	[25–32]
Avian influenza A (H5N1)	Posterior‐pharyngeal (throat) swab, nasal swab, tracheal aspirates, autopsy tissues (lung, brain, heart, spleen, liver, kidney, large intestine, and small intestine)	[33, 34]
Ebola	Whole blood, oral swab, seminal fluid, saliva, stool, breast milk, tears	[35–37]
Zika	Serum, plasma, urine, whole blood, amniotic fluid, cerebrospinal fluid (CSF), placenta, brain, saliva, semen, vaginal discharge, sweat, breast milk	[38–42]
Dengue	Serum, plasma, blood, CSF, breast milk	[43–46]
West Nile	Homogenized tissues, serum, whole blood, urine, CSF	[47–50]
Japanese encephalitis	Brain tissues, throat swab	[51, 52]
Enterovirus 71	Serum, vesicle swab; CSF, heart and lung tissues, stool, serum, rectal swab, nasopharyngeal swab, throat swab, eye swab, and intestinal, lymph node, and tonsillar tissues.	[53, 54]
Coxsackievirus	Throat swab, rectal swab, vesicle swab, ulcer swab, conjunctival swab, serum, CSF	[53, 55–57]
Chikungunya	Whole blood, CSF	[58, 59]
Rabies	Saliva, CSF, serum, blood, urine, brain tissues, neck skin tissues	[60]

The standard sample type for RNA extraction to detect SARS‐CoV‐2 is a nasopharyngeal swab. This procedure requires trained medical personnel, because it carries an infection risk due to aerosol generation during sample collection. Additionally, collecting nasopharyngeal swabs can cause discomfort and bleeding [30]. Saliva, a noninvasive sample, has been used as an alternative sample type for SARS‐CoV‐2 detection. Saliva collection procedure is relatively simple, thereby reducing the risk of infection to healthcare workers and eliminating the need for trained healthcare workers. In addition, the use of saliva samples could facilitate SARS‐CoV‐2 detection in remote and low‐resource settings [27, 28, 30]. The sensitivity of saliva samples to detect SARS‐CoV‐2 ranges between 78% and 100% [27]. Recently, self‐collected gargle specimens have been proposed as a user‐friendly sample collection technique for SARS‐CoV‐2 detection [31]. Although saliva and gargle are appealing alternatives to nasopharyngeal swabs, several challenges have been reported. The methods for sample collection and processing are not standardized, and conflicting results have been observed when comparing viral loads between saliva and nasopharyngeal samples. Additionally, there are concerns about higher false‐negative rates in viral detection using saliva and gargle samples. Advantages, challenges, and advances in techniques for collection, processing, RNA extraction, RNA preservation of saliva, and gargle samples have been reviewed elsewhere [61].

The types of specimens suitable for molecular detection of influenza viruses include nasal, throat, oropharyngeal, and nasopharyngeal swabs. Nasopharyngeal or bronchial aspirates can also be used [34]. The risk of false negatives is increasing when dealing with low viral load samples due to an accelerated degradation of RNA, as noted in an influenza virus surveillance study [62]. On the other hand, not all droplet‐borne viruses can be optimally detected in respiratory tract specimens. For example, respiratory tract specimens have lower sensitivity compared to blood specimens for detecting Ebola virus (EBOV) by nucleic acid amplification test (NAAT). In situations where blood collection is not feasible, such as with children or deceased patients, oral swabs stored in a universal transport medium can be used [36]. It is recommended to collect specimens for RNA extraction from EBOV‐suspected patients as soon as symptoms are observed. Specimens can be stored at room temperature for up to 24 h. For long‐term storage, it should be kept at −70°C, while avoiding freeze–thaw cycles [36]. Arthropod‐borne viruses or arboviruses, notably CHIKV, DENV, WNV, and ZIKV, are pathogens of public health significance. Clinically distinguishing between viruses particularly during the acute phase of infections is often challenging. Therefore, methods with better sensitivity and specificity, such as NAAT, are necessary. Since arboviruses primarily replicate in the blood circulatory system, the recommended specimen types for virus detection are whole blood, serum, or plasma [14]. In most cases, the virus infection is acute but self‐limiting, making the timing of specimen collection critical for optimal detection of viral RNA. Viremia is typically detectable in blood in the first 5 days after symptom onset [42, 48, 50, 58, 63]. Moreover, urine is an alternative specimen type that can be used to detect the viruses, particularly in the later stage of infection [40, 49, 50, 64]. Other specimen types have also been reported to successfully detect arbovirus RNA, albeit with varying sensitivity and specificity: amniotic fluid [41], cerebrospinal fluid (CSF) [14, 46, 50, 59], fetal brain and placenta [38], saliva [40, 64], throat swab [51], oral swab [65], vaginal secretions [66], sweat, semen, rectal secretions [67], and breast milk [45, 68].

JEV, another significant arbovirus, is a major cause of central nervous system infections in Asia [69]. This zoonotic virus uses swine as a reservoir host and mosquitoes, primarily *Culex tritaeniorhynchus* as the main vector [70]. In humans, viral RNA is rarely detected in CSF or serum, although it has been documented in throat swab specimens [51]. For fatal cases, brain samples may be utilized for virus detection [52].

During the hand, foot, and mouth disease (HFMD) outbreak in Singapore in 2000, molecular detection of enterovirus 71 (EV‐71) was performed on multiple types of specimens from both live and deceased pediatric patients. From live patients, EV‐71 RNA was detected in specimens such as throat swabs, stools, nasopharyngeal aspirates, vesicle swabs, mouth ulcers, oral swabs, and rectal swabs. From the deceased patients, EV‐71 RNA was detected in specimens such as rectal swabs, foot swabs, saliva, lung, heart, brain, tracheal swabs, spleen, oral swabs, stool, and tonsils [71]. Throat swabs collected from pediatric patients suffering HFMD, herpangina, and nonspecific febrile illness had been used for EV‐71 molecular detection [72]. Similarly, various types of samples have been utilized for the detection of EV‐71 RNA, including from a sibling of HFMD fatal case in Jakarta, Indonesia [54]; patients with HFMD in Shanghai, China [73]; molecular detection efforts in Africa and Madagascar [74]; in acute flaccid paralysis cases in Senegal [75]; and during the HFMD outbreak in Cambodia in 2012–2013 [76].

For molecular detection of coxsackievirus, serum and swabs from the throat, rectum, vesicle, and ulcer are suitable samples, as coxsackievirus A16 has been successfully isolated from the sample types. Throat swabs have been found to yield the highest positive results for virus isolation [53]. Conjunctival swab specimens collected from patients with acute hemorrhagic conjunctivitis were used to detect coxsackievirus A24 RNA in India [55]. In Indonesia, coxsackievirus B3 RNA was detected in serum specimens collected from a patient with undifferentiated febrile illness [56]. In the West Bank, Palestine, coxsackievirus B5 RNA was detected in CSF samples from patients with suspected sepsis‐like illness and/or aseptic meningitis [57]. Additionally, coxsackievirus A6 RNA has been reported in formalin‐fixed, paraffin‐embedded (FFPE) skin biopsy specimens [77].

The measles virus is highly contagious, and nosocomial transmission is a common issue. The virus is also detected in air and surface specimens in a hospital environment [78]. For the detection of measles viral RNA, nasal swabs were collected for RNA recovery from patients with suspected measles in an outbreak occurring in children in Banjarmasin, Indonesia, in 2014 [79]. Moreover, rabies is a fatal zoonotic disease, the vast majority of which is transmitted to humans through dog bites [80]. Types of samples that can be employed for the detection of rabies viral RNA include saliva, CSF, serum, blood, and urine for ante‐mortem diagnosis, and brain and neck skin tissues for postmortem investigation. It was reported that the saliva specimen gave the highest number of positive test results [60]. Rabies virus RNA can also be detected in dog brain tissue specimens [81, 82]. For hepatitis A and E, which are transmitted via the fecal–oral route, and hepatitis C, which is transmitted through parenteral means, RNA extraction platforms must be carefully selected based on the type of virus and nature of the specimen [83].

### 2.2. Sample Processing, Storage, and Shipment

Apart from the sample type, successful RNA isolation also depends on the time of sample collection (stage of infection), collection methods, and proper specimen handling. Samples should be processed immediately after collection, as RNA viruses are generally less stable outside the host body and easily inactivated by heat. However, immediate processing is often impractical in many settings. Collected samples should be stored at low temperatures, such as on ice, in a refrigerator, or in a freezer, to preserve their integrity until further processing. For long‐term storage, specimens should be kept frozen at −20°C or, ideally, at −70°C [84]. For shipping, specimens should be placed in a suitable transport medium and protected from thermal inactivation [24, 84]. In general, proper handling of viral specimens from collection to transportation is a key to extract high‐quality RNA.

For successful viral RNA extraction and subsequent molecular analysis, appropriate sample processing is critical, including collection, on‐site virus inactivation, RNA preservation, RNA extraction and concentration, and the removal of inhibitors that may interfere with nucleic acid amplification [61]. In resource‐limited or field settings, safe handling of infectious RNA virus samples remains a major challenge. A method such as magnetic‐nanoparticle‐aided viral RNA isolation from contagious samples (MAVRICS), based on phenol–chloroform‐mediated inactivation, has been developed to reduce biohazard risk and simplify [85]. Viral transport medium (VTM) plays a critical role in maintaining virus integrity during specimen transport. The medium is typically composed of a buffer for pH maintenance, a protein stabilizing agent, and antimicrobial agents [86]. For virus isolation, specific types of VTM are formulated to preserve viral activity and sustain viral replication capacity [86, 87]. In contrast, for accurate molecular diagnosis, VTM may be designed to inactivate viruses while preserving viral RNA, making it unsuitable for virus culture. A recent review has detailed the composition, usage, and performance of various types of VTM [86]. Additionally, large‐scale, in‐house VTM production methods have been proposed to address potential shortages, particularly during outbreaks or pandemics [88].

Buffer AVL (QIAGEN) is commonly employed in the storage of RNA viral samples. This buffer contains chaotropic salt guanidinium thiocyanate, which denatures macromolecules and protects viral RNA during the extraction process. Detergents, such as Triton X‐100, have been used for viral inactivation prior to RNA extraction. It has been reported that the addition of Triton X‐100 in buffer AVL does not interfere with RNA extraction quality [89]. Viral RNA in samples stored at room temperature may degrade rapidly, and repeated freeze–thaw cycles during cold storage can also compromise RNA integrity [89]. Conversely, a study using SARS‐CoV‐2‐positive samples found that viral RNA remains relatively stable after sample storage in VTM produced according to the protocol of the Institute of Medical Virology, University of Zurich, Zurich, Switzerland, for 21 days at higher temperatures (25°C or 35°C), even with up to 15 freeze–thaw cycles [90]. Recent research has investigated the stability of SARS‐CoV‐2 RNA in wastewater samples under various conditions, including time, temperature, and freeze–thaw cycles. To maintain viral RNA stability across various storage temperatures (−80°C, −20°C, 4°C, or 20°C), it is recommended that wastewater samples be stored at or below 4°C after sample collection and that viral extraction be performed as promptly as possible [91]. Nevertheless, harmonizing these protocols across laboratories remains difficult. For instance, VTM optimized for viral culture differs from those tailored for molecular detection, complicating downstream research workflows.

For shipment, clinical specimens must be properly packaged, labeled, marked, and documented [36]. It is important to recognize that shipping infectious viral samples carries a high risk of environmental contamination due to potential spillage during transport. Additionally, transporting viral samples over long distances requires cold‐chain facilities to prevent temperature fluctuations that could compromise sample integrity. In resource‐constrained settings, maintaining stringent cold‐chain conditions can be challenging. To address these logistical and infrastructural constraints, alternative cold chain–independent specimen collection and transport medium have been developed to facilitate timely diagnosis in developing countries. Notably, this cold chain–independent transport medium has been reported to offer improved diagnostic sensitivity compared to commercial VTMs [92]. Alternatively, Flinders Technology Associates (FTA) classic cards can be used to transport viral specimens at ambient temperature over extended periods. These cards contain chaotropic agents that preserve viruses and stabilize RNA molecules [93]. Similarly, the RNA*Sound* sampling card has been designed to inactivate viruses while preserving viral RNA, enabling safe transport of RNA samples. This card can maintain viral RNA stability at ambient temperature for up to 1 month [94]. Commercial RNA preservatives such as OMNIgene‐GUT and Zymo DNA/RNA shield [95], along with various storage matrices [96], have also been evaluated. Moving forward, integrating these novel transport and preservation solutions with automated RNA extraction and molecular detection platforms will be essential to enable seamless workflows, particularly for point‐of‐care (POC) applications in low‐resource environments.

## 3. Principles of Viral RNA Extraction

**Figure 2:**
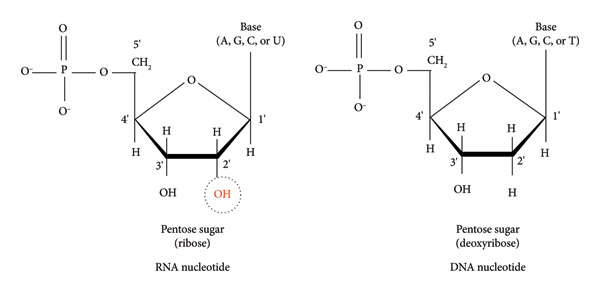
Chemical structures of RNA nucleotide and DNA nucleotide. RNA is much less stable than DNA due to the presence of 2′‐hydroxyl group on the 2′ carbon of the pentose ring, which increases the RNA molecule’s susceptibility to hydrolysis.

RNA extraction is a crucial step in the molecular detection and characterization of RNA viruses. It serves as the starting point for a myriad downstream applications, including the development of molecular diagnostics and disease countermeasures [97]. Extracting RNA from viruses presents challenges due to RNA instability and the infectious nature of the virus [98]. RNA is inherently less stable than DNA due to the presence of a 2′‐hydroxyl group on the 2′ carbon of the pentose ring (Figure 2) that is more susceptible to hydrolysis [99]. The instability is further exacerbated by the pervasive and resilient nature of RNA‐degrading enzyme, ribonuclease (RNase) in the environment. Therefore, it is essential to develop strategies to protect RNA molecules from enzymatic degradation. Commonly used RNase inhibitors include vanadyl ribonucleoside complexes (VDR), RNasin, and diethyl pyrocarbonate (DEPC) [99].

Virus is an obligate cellular parasite; thus, viral RNA isolation requires specific strategies to remove host cell contaminants, such as DNA, proteins, polysaccharides, and lipids [100]. Additionally, viral RNA is typically present in much lower quantity compared to host RNA, making methods, such as host rRNA depletion, beneficial for improving viral RNA recovery [101]. In general, viral RNA extraction from biological samples involves four key steps: disruption of tissue and cellular structures, denaturation of nucleoprotein complexes, inactivation of ribonucleases, and purification of RNA [97]. The grinding step is involved for tissue disruption and homogenization [47]. Common techniques for releasing viral RNA involve heating, osmotic shock, detergents, chaotropic salts, and organic solvents, which can be used alone or in combination [100]. In addition to in‐house reagents, standardized reagents for RNA extraction are available in quality‐controlled commercial kits [97]. The efficiency of RNA extraction can be improved by employing deoxyribonuclease (DNase) to remove contaminating DNA molecules [102]. Several methods have been developed for efficient viral RNA extraction. The three widely used methods are organic extraction (TRIzol‐based), spin column–based, and magnetic bead–based methods [103]. Recently, robotic RNA extraction platforms have been introduced to enhance extraction speed [104].

### 3.1. Organic Extraction

**Figure 3:**
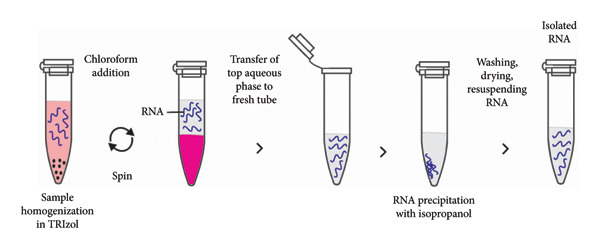
The TRIzol‐based RNA extraction method. TRIzol reagent contains phenol and guanidium thiocyanate under acidic conditions. Phenol extracts nucleic acids and proteins, while the guanidium salt functions as a chaotropic agent to denature proteins. The acidic environment separates RNA from DNA and protein. Adding chloroform induces phase separation, with RNA in the upper aqueous phase, protein in the lower organic phase, and DNA at the interface. RNA is then collected from the upper aqueous phase, precipitated using isopropanol, washed with ethanol solution, dried, and resuspended in a buffer solution.

TRIzol‐based organic extraction method uses TRIzol reagent to extract RNA molecules from various biological samples. The reagent contains phenol and guanidium thiocyanate in an acidic condition. Phenol is a common organic compound for nucleic acid and protein extraction, while guanidium salt functions as a chaotropic agent for denaturing proteins. The acidic condition is critical for separating RNA from DNA and protein because at high pH, RNA and DNA molecules would stay together [105]. At low pH, double‐stranded DNA tends to denature and the resultant single‐stranded DNA partitions to phenolic solvent [106, 107]. Moreover, at acidic pH, the RNA phosphodiester bond is more stable and RNA degradation is minimized. On the other hand, the RNA molecule is susceptible to alkaline hydrolysis at high pH [108]. TRIzol reagent is a monophasic solution that simultaneously solubilizes biological material and denatures protein. Upon adding chloroform after the solubilization process, phase separation occurs. RNA partitions into the upper aqueous phase, proteins move into the lower organic phase, and DNA remains at the interface (Figure 3) [109]. The RNA in the upper aqueous phase is then collected, precipitated with isopropanol, washed with ethanol, dried, and resuspended in a buffer solution [105, 109]. The optimized phenol–chloroform‐based RNA extraction method can be supplemented with proteinase K and guanidium‐based lysis buffer to break down cell walls or organelles effectively [110]. The method is widely used in many laboratories worldwide and yields comparable results to automated systems for diagnosing RNA viruses, such as SARS‐CoV‐2. Thus, it is a viable alternative to automated systems when proprietary materials are inaccessible [111]. Compared to commercially available RNA extraction kits, phenol–chloroform RNA method is more economical and often provides better RNA yield, particularly when extracting RNA from small quantities of cells or tissues. However, this technique is prone to contamination from excess phenol, guanidine, chloroform, and salts, which may interfere with downstream applications, such as RNA quantification and reverse transcription [112]. It is essential to adhere to biosafety guidelines when processing specimens associated with pathogenic viruses [113, 114]. Notably, guanidium thiocyanate‐containing lysis buffer can render highly pathogenic viruses safe for handling in a biosafety level 2 laboratory [89].

### 3.2. Spin Columns

**Figure 4:**
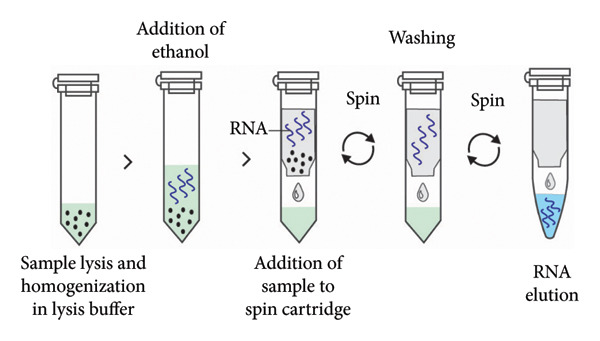
Silica column–based RNA extraction method. The sample is initially lysed and homogenized in lysis buffer containing a chaotropic agent such as guanidinium thiocyanate which releases the RNA. Ethanol is then added to facilitate RNA adsorption to the silica surface. The sample lysate in binding solution is transferred to a spin cartridge, followed by centrifugation. The column is washed by adding a washing buffer followed by centrifugation, and the viral RNA is finally eluted from the column using an elution buffer.

**Figure 5:**
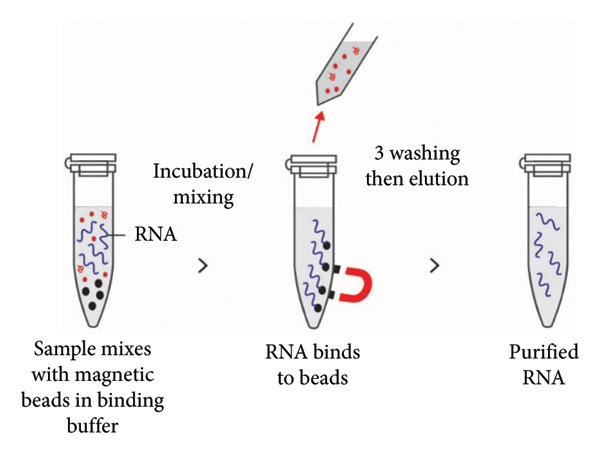
Magnetic beads–based RNA extraction method. The lysed sample is incubated with magnetic beads to allow the binding of RNA molecules to the magnetic particles. The viral RNA‐containing magnetic beads are collected by placing them near an external magnetic field. Following removal of supernatant and washing of magnetic beads, the viral RNA is eluted from the magnetic beads using RNase‐free water or an elution buffer.

Spin column–based method is a solid‐phase RNA extraction method that utilizes RNA binding to silica particles, glass particles, diatoms, or ion exchange carriers (Figure 4). The interaction between negatively charged nucleic acids and positively charged silica results in the selective binding of nucleic acids to the silica matrix, allowing for the removal of remaining cell components and contaminants [107]. This method consists of four main steps: lysis, binding, washing, and elution. First, the sample is lysed using a lysis buffer containing chaotropic agents such as guanidinium thiocyanate, which helps to release RNA and inactivate RNases to prevent RNA degradation. The lysate is then transferred to a spin column containing binding solution with a specific pH and salt concentration optimized for RNA binding to the column matrix. During binding, non‐RNA molecules pass through the column, while RNA adheres to the matrix. The addition of ethanol or isopropanol during RNA adsorption enhances RNA binding by reducing its solubility [115]. Following binding, RNA is washed with washing buffer to remove salts and contaminants. Vacuum‐based systems have been used as an alternative to centrifugation to remove impurities. Finally, RNA is eluted from the column using elution buffer (Figure 5) [107, 116–118].

Silica column–based viral RNA purification methods are widely utilized due to their ability to rapidly isolate high‐yield and quality viral RNA. Most commercial kits employ guanidinium salt‐based cell lysis buffer without toxic organic solvents, followed by RNA purification using a silica column. However, these techniques are often expensive and offer limited flexibility [119]. To address cost concerns, one proposed strategy is to reuse the silica column. A study has shown that a silica column can be reused up to five times. Regeneration involves treating the columns with a warm alkaline solution containing Triton X‐100 to remove residual RNA. This approach is particularly valuable in situations where there is a shortage of commercial silica column–based viral RNA extraction kits [120].

### 3.3. Magnetic Bead

**Figure 6:**
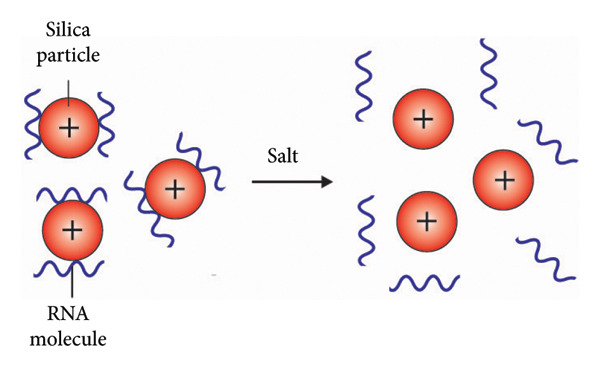
The binding of RNA molecule to silica particles. The affinity between negatively charged RNA molecules and positively charged silica particles causes the selective binding of RNA to silica matrices.

Magnetic bead–based method utilizes coated iron oxide particles, with binding specificity determined by surface coating chemistry. Under solid‐phase conditions, the coated beads can reversibly bind nucleic acids. The lysed sample is incubated with the magnetic beads, allowing binding of RNA molecules to the magnetic particles. The RNA‐containing magnetic beads are then immobilized using a strong magnet, facilitating repeated washing and manipulation steps. Following removal of the supernatant and washing the magnetic beads, RNA is eluted using RNase‐free water or elution buffer (Figure 6) [118, 121]. This method is cost‐effective and does not require centrifugation, making it easily scalable for processing more samples per extraction batch. Additionally, it is simple to handle and can be automated [118, 121, 122].

In‐house rapid and field‐compatible viral RNA extraction platforms based on silica‐coated magnetic beads have been developed and demonstrated to have comparable sensitivity and specificity with commercially available viral RNA extraction kits. These user‐friendly, cost‐effective methods are proposed to be suitable for on‐site detection of RNA viruses such as Chikungunya [123], SARS‐CoV‐2 [124, 125], and Kyasanur forest disease virus [126] in resource‐limited settings. A large‐scale RNA extraction method utilizing magnetic beads has been developed for viral RNA isolation. This method employs silica‐coated magnetic beads, which are easy to produce and sustainable. Studies have reported that viral RNA yield using this method is comparable to commercial kits [124] and more cost‐effective [127]. Of note, a simple one‐pot process employing reverse micelles as nanoreactors has been developed for synthesizing uniformly sized silica‐coated magnetic nanoparticles [128]. The surface of the silica‐coated magnetic nanoparticles can be modified to improve binding to viral RNA and therefore give better yield of viral RNA extraction from samples [129]. To reduce dependency on commercial kits, open‐source magnetic bead–based methods for nucleic acid extraction and manipulation have been proposed. Detailed protocols for synthesizing magnetic bead and their application for RNA extraction and other relevant processes have been described elsewhere [122].

## 4. Optimization of Viral RNA Extraction

Optimization of the RNA extraction process is critical for obtaining high‐quality and high‐quantity viral RNA. This can be achieved by reducing processing time, safety risks, and costs [130]. Factors that can be optimized include lysis buffer concentration, extraction conditions, magnetic bead quantity, RNA stability, and reagent availability [117, 130, 131]. No single RNA extraction method is suitable for all types of specimens, making it essential to understand the sample type and viral characteristics before selecting the most appropriate RNA extraction technique [132–134]. In order to improve speed, capacity, and RNA concentration, robotic RNA extraction platforms have been developed [104, 135]. These methods have been tested for detecting hepatitis A virus in contaminated bottled water [135] and SARS‐CoV‐2 in human saliva samples [104].

The “gold standard” for viral RNA preservation is freezing it at an ultra‐low temperature of −70°C. However, a notable study found that viral RNA integrity can be maintained at room temperature for up to 16 weeks by placing the RNA on spin columns [117]. Another method for maintaining RNA stability at ambient temperature involves storing RNA on a glass microfiber (GF/D) membrane for up to 35 days [136]. Additionally, RNA can be preserved for up to 92 days at an elevated temperature of 45°C in a dry matrix, such as RNAstable [137]. These simple and economical RNA storage techniques are valuable for molecular detection of RNA viruses as well as outbreak responses in resource‐limited settings.

Optimization of commercial viral RNA extraction kits can be achieved by modifying the manufacturer’s procedure. Adjustments such as prolonged ethanol evaporation time, elution incubation time, and centrifugation steps have been shown to increase RNA recovery, thereby enhancing the sensitivity of viral RNA detection [138]. Moreover, the emergence of SARS‐CoV‐2 variants, such as the Omicron variant, increased the viscosity of mucus and turbidity of the clinical specimens, interfering with the viral RNA extraction process and affecting the diagnostic accuracy. To address this, a modification was made by diluting samples with saline solution at a 1:2 ratio before RNA extraction [139]. Additionally, viral RNA recovery from plasma or other body fluids with low viral loads can be improved by pelleting viral particles in siliconized microcentrifuge tubes [140]. For efficient viral RNA recovery from insect vectors, different methods have been developed. For instance, a combination of QIAzol, proteinase K treatment, and silica magnetic beads could significantly improve viral RNA recovery from insects and was suggested as a useful technique for arbovirus molecular surveillance [141]. An optimized phenol–chloroform‐based RNA extraction method has also been successfully employed to extract DENV RNA from FFPE tissues [110]. Extracting viral RNA from tissue or swab samples is considered more challenging than from cell lysates or cell culture supernatants due to their complex composition. While any RNA extraction method can be applied to swab samples, it is recommended to use organic extraction methods for tissue samples following the preparation of a 10% (w/v) homogenate in phosphate‐buffered saline [142].

Coinfections of viral and bacterial pathogens often occur in clinical settings, making timely molecular pathogen detection critical for effective treatment. This necessitates a specialized extraction method that allows for simultaneous isolation of viral and bacterial nucleic acids for molecular diagnosis [143]. A simple, rapid, and reliable technique for simultaneous extraction of viral RNA and bacterial DNA from clinical samples has been evaluated [143]. This method is a modified bacterial DNA extraction protocol that involves cell lysis, DNA adsorption onto silica membrane with the addition of carrier RNA, and the use of a centrifugal filter to improve viral RNA recovery. One of the problems associated with the simultaneous processing of bacteria and viruses is the difference in the protective layers surrounding their nucleic acids. Generally, bacteria are tougher to lyse than viruses. To ensure efficient lysis of Gram‐positive and Gram‐negative bacterial cell walls, this method involves bead‐beating and lysozyme treatments [143]. This approach reduces the sample preparatory work, cost, time, labor, and resources compared to multiple extraction procedures [143].

Optimized magnetic bead–based RNA extraction methods using readily accessible reagents have been developed to minimize cross‐well contamination without compromising sensitivity [144]. A simple magnetic bead–based viral RNA extraction technique for rapid and high‐throughput RNA extraction suitable for various settings has been proposed [145]. Recently, an efficient and readily automatable viral RNA extraction method utilizing locally synthesized silica‐coated magnetic microparticles has been developed and optimized to achieve high yield and purity of viral RNA. This method performs comparably to commercial magnetic nanoparticle methods and is potentially suitable for local high‐throughput sample processing where consumables are limited [146]. Additionally, in order to efficiently detect both viral RNA and DNA in a single clinical sample, a method for coextraction of viral RNA and DNA has been developed [130]. This magnetic bead–based method was found to perform best using a lysis buffer composed of 2 M guanidinium thiocyanate, 80 mM dithiothreitol, RNA carrier, and 200 μg magnetic beads at pH 8–9, followed by incubation at 80°C. This established method has the potential to reduce time, cost, and risk of exposure to harmful chemicals [130].

Development of in‐house viral RNA extraction methods can enhance sample processing capacity and ensure the continuity of viral RNA isolation capabilities during commercial diagnostic kit shortages, such as in a global pandemic setting. Sharing various viral RNA extraction methods among laboratories is highly encouraged in these situations [138]. To address reagent shortages, an in‐house viral RNA isolation method using homemade lysis buffer and washing solution has been created [131]. The unstable nature of RNA often poses challenges for isolation and detection, particularly in remote areas and underdeveloped regions. To meet the requirements for field viral RNA extraction and detection, a simplified and inexpensive RNA extraction device was created using a syringe and a stable denaturing buffer. The device consists of a plastic cap attached to a standard syringe, an elution tip, a gasket O‐ring, and an RNA‐binding membrane [147].

The efficiency of viral RNA extraction can be improved by incorporating carrier RNA [148, 149]. Carrier RNA modulates RNA capture and release during the RNA extraction process, playing a critical role in optimizing RNA yield. Recently, a polyplex‐based viral RNA extraction carrier composed of cationic poly (2‐(dimethylamino)ethyl acrylate) electrostatically conjugated with RNA was developed [149]. Carrier RNA also functions as a preservative stabilizing and protecting RNA, especially from RNases during extraction. Yeast RNA has been employed as a carrier for viral RNA extraction due to its abundance, stability, and resistance to RNase degradation [148]. The use of linearized polyacrylamide (LPA) as a carrier to improve RNA yield has also been evaluated. Notably, most commercial RNA extraction kits use relatively expensive polyadenylate (poly A) carrier RNA to reduce RNA loss and improve yield. The advantages of the LPA as a carrier include low cost, easy preparation, efficient precipitation of low concentrations of viral RNA with ethanol, and no interference with downstream applications [131].

Clinically important RNA viruses, such as SARS‐CoV‐2, can be detected in fecal samples and contaminated wastewater. Monitoring viral prevalence, spread, and evolution in wastewater is crucial, necessitating an effective method for isolating viral RNA from these samples [150]. A cost‐effective, safe, and reproducible method for isolating viral RNA from wastewater has been developed using TRIzol. This method involves sample pasteurization at 60°C for 1 hour prior to RNA extraction to inactivate viruses. The pasteurization process has been shown to preserve viral RNA integrity and enhance viral RNA recovery by facilitating the release of viruses bound to wastewater solids [150]. In wastewater surveillance, effective and optimized viral RNA isolation methods are required for monitoring low viral concentrations of pathogenic RNA viruses, including sample concentrating methods [151]. Ultrafiltration for concentrating viral particles from influent samples combined with silica bead–based extraction and neutral phenol–chloroform treatment was demonstrated to be effective for detecting pathogenic RNA viruses in wastewater [151]. In addition, a study conducted in Central Italy found that SARS‐CoV‐2 RNA was detected in swabs collected on environmental surfaces, such as toilet areas, snack and drink vending machines, frequently touched handles, and large classrooms. The viral RNA was extracted from swab specimens using a total RNA purification kit [152].

The efficiency of viral RNA extraction methods is crucial for detecting food‐borne pathogenic viruses, such as the norovirus and hepatitis A in fruits and vegetables [153]. Norovirus is the leading cause of acute viral gastroenteritis worldwide, resulting in over 1.8 million child deaths each year [154]. Similarly, hepatitis A virus is a major contributor to viral food‐borne infection outbreaks [155], with 159 million cases reported globally in 2019 and over 39,000 deaths [156]. Viral RNA extraction from fruit and vegetable samples is challenging due to the presence of organic and inorganic substances in complex matrices that may act as inhibitors in subsequent applications. Additionally, the variability in composition, volume‐to‐weight ratios, quality, ripeness, previous treatment, and storage of different fruits and vegetables poses challenges in the development of a universal RNA extraction method [153]. An optimized protocol for viral RNA extraction from fruits and vegetables has been developed involving direct lysis of viral capsid after viral elution from food matrices that can improve viral RNA recovery [153]. Efficient viral RNA extraction can be achieved by increasing the lysis buffer volume to submerge the samples. The use of pectinase, a plant RNA isolation aid and PCR inhibitor removal kit, has been applied to increase the efficiency of inhibitor removal, potentially improving the detection of pathogenic RNA viruses in foods of plant origin [153].

One of the challenges in applying next‐generation sequencing (NGS) to sequence viral RNA genomes is the relatively low abundance of viral RNA compared to that of host nucleic acids [101]. To address this, a methodology was established to increase the percentage of viral RNA by depleting the host genomic DNA and rRNA. The strategy has been successfully applied for obtaining the full genome of lyssaviruses and is potentially applicable to other RNA viruses [101].To facilitate purification of viral RNA in its native form and remove host RNA background, a capture‐based purification method for viral RNA has been designed involving hybridization, capture, washes, de‐hybridization, and clean‐up steps. This approach can be integrated with downstream applications such as direct RNA sequencing [157]. For rapid whole genome sequencing of various RNA viruses, a technique to remove nonviral DNA and RNA using nuclease treatment has been developed [158]. The method has been demonstrated to exhibit 100% accuracy and can potentially be applied for cost‐effective, accurate determination of viral RNA genomes using portable DNA sequencers, e.g., Oxford Nanopore MinION [158]. Future optimization should prioritize not only yield and purity but also compatibility with multiplexed PCR and sequencing workflows and developing protocols suitable for difficult matrices, such as wastewater, FFPE tissues, and environmental swabs. Addressing these integration challenges will be key to ensuring that novel RNA extraction platforms can meet both research and diagnostic needs.

## 5. Comparison of RNA Extraction Methods

Effective RNA isolation aims to provide viral RNA of high purity and integrity for downstream molecular applications [103]. It is essential to select the most suitable commercial RNA extraction kit, as different kits yield varying results in terms of quantity and quality of viral RNA, which may affect the subsequent analyses such as NGS and quantitative reverse transcription‐polymerase chain reaction (qRT‐PCR) [132]. In general, key factors to consider when selecting viral RNA extraction methods and kits include extraction efficiency, RNA purity and integrity, RNA yield, downstream processes, speed, sample types, reliability and repeatability, cost‐effectiveness, availability, flexibility, scale, simplicity, portability, and safety [102, 103, 132, 136, 145, 147, 159]. A study comparing four different commercially available RNA extraction kits showed that a kit based on the spin column technique was superior in terms of RNA quantity and quality, while one using the magnetic bead method was faster [103]. However, for mRNA extraction of human papillomavirus, magnetic bead–based methods were demonstrated to be superior to column‐based methods [159].

A study comparing manual and automated RNA extraction methods for SARS‐CoV‐2 detection found no significant differences in results by qRT‐PCR. However, there were notable differences in time, labor, and cost. The manual method needed twice as much labor and took 40 min longer. In terms of cost, the automated method was 3.5 times more expensive for consumables but gave better yield [160]. To reduce labor, time, waste, and cost, a semiautomated viral RNA extraction protocol was developed by replacing the centrifugation steps of a spin‐based extraction method with vacuum filtration. This approach allows for a quicker extraction process and accurate quantitative assay results, making it a viable alternative for use in developing countries due to its efficiency and ease of use [161]. Other comparison studies showed that both commercial manual and automated viral RNA extraction kits have distinct advantages and drawbacks, and both can be employed for molecular detection of RNA viruses [162]. For example, the amount of viral RNA isolated using a manual extraction kit (GF‐1, Vivantis) was found to be significantly higher compared to an automated extraction kit (Nextractor NX‐48S, Genolution), although both produced viral RNA of comparable purity. However, the automated kit was able to process more samples in a much shorter time [162].

Three different viral RNA extraction techniques, the phenol/chloroform method, the RTP DNA/RNA virus mini kit, and the automated MagNa PureLC (MPLC) extraction, were compared for their capacity in isolating respiratory viral RNA from patient sputum samples [163]. Results showed that the MPLC method gave the best results [163], though sputum is challenging due to the high levels of ribonucleases [163]. For direct molecular detection of poliovirus in stool samples, the performance of 11 RNA extraction kits was compared [133]. Results showed that the MagMAX viral RNA Isolation Kit was considered the best overall based on performance, simplicity, cost, and flexibility [133].

For molecular diagnosis of RNA viruses in field settings, the performance of four simple RNA extraction methods was compared [164] using plasma samples. Both silica‐membrane‐based and magnetic beads–based methods exhibited a 100% detection rate with real‐time RT‐PCR. Using reverse transcription loop‐mediated isothermal amplification (RT‐LAMP), the detection rates dropped to 66% and 62%, respectively. The magnetic beads–based extraction method is adaptable, rapid, affordable and does not involve a centrifugation step and was recommended as an alternative method for viral RNA extraction from plasma in field settings [164]. Wastewater‐based epidemiology of RNA viruses is increasingly utilized to monitor viral spread. A study comparing four commercial RNA extraction kits has been carried out [165], showing that the QIAGEN viral kit consistently recovered the greatest number of viral RNA copies followed by the Promega, IDEXX, and QIAGEN Water kits [165]. Overall, the key challenge is to balance speed, biosafety, and affordability, and downstream compatibility. Future work should prioritize hybrid approaches that combine the robustness of commercial kits with the flexibility and cost‐effectiveness of novel methods to ensure broad applicability in both diagnostic and research settings.

## 6. Application of Extracted Viral RNA

**Figure 7:**
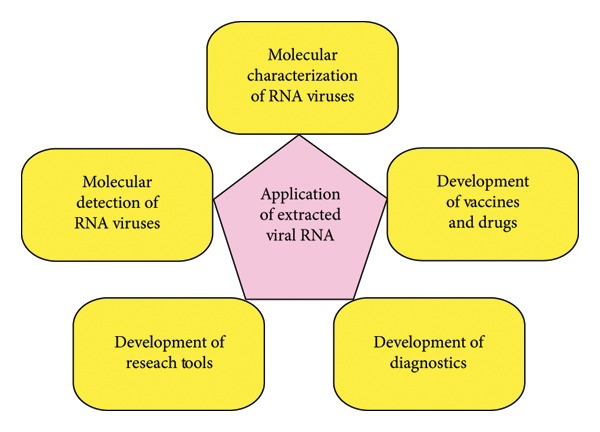
Ranges of application of extracted viral RNA. Extracted viral RNA is utilized in various applications, including molecular detection and characterization of RNA viruses, development of vaccines and drugs against RNA viruses, development of diagnostic tools for RNA viruses, and development of research tools for the molecular study of RNA viruses.

Extracted viral RNA has a wide range of applications, especially for outbreak response strategies and molecular characterization studies. High‐quality isolated viral RNA is essential for molecular detection and characterization of RNA viruses [166]. In addition, viral RNA also plays a critical role in the development of vaccines [167], drugs [168], diagnostics [169, 170], and research tools [171], as illustrated in Figure 7.

PCR‐based techniques together with DNA sequencing methods provide highly sensitive and specific approaches for detecting and identifying pathogenic viruses. The real‐time PCR platform, for example, has been considered as the “gold standard” for SARS‐CoV‐2 detection during the COVID‐19 pandemic [172]. For detecting RNA viruses using PCR‐based strategies, viral RNA is first reverse transcribed into complementary DNA (cDNA), which then serve as a template for subsequent PCR amplification. The resulting amplicon can be subjected to nucleotide sequence analysis for further viral identification. RT‐PCR‐based procedures have been employed to detect the circulation of various RNA viruses, such as influenza viruses [173–175], EBOV [176], ZIKV [177–179], SARS‐CoV‐2 [180, 181], DENV [43], CHIKV [63], WNV [48], JEV [51], coxsackievirus [56], measles virus [79], EV‐71 [166], and rhinovirus C [182].

The next frontier of application is the integration of extraction technologies into portable POC devices. An ultracompact device combining plasma separation, viral RNA extraction, and nucleic acid analysis has been designed to facilitate regular viral load monitoring in POC settings [183]. This device employed a centrifugation‐independent syringe‐based viral RNA extraction module, providing an extraction efficiency for on‐site plasma processing of about 86% [183]. Similarly, a portable, user‐friendly device integrating viral RNA extraction with nucleic acid amplification has been designed to allow rapid molecular diagnosis [184]. In this system, viral particles are captured on an RNA extraction membrane and lysed with buffer, and the extracted RNA is subsequently eluted onto a reaction membrane for downstream analysis. Other novel approaches include a POC test that enables RNA virus detection directly from saliva using a semi‐alkaline proteinase‐based RNA extraction method [185]. Of note, to improve cost‐effectiveness, an open platform of POC technologies capable of simultaneously detecting multiple RNA viruses is important [186]. A POC testing for RNA viruses which integrates viral RNA extraction with PCR has been developed. In this system, viral RNA captured by magnetic beads is washed and then released into a PCR chip for subsequent analysis [186]. Future challenges involve developing modular, cost‐effective POC platforms capable of detecting multiple RNA viruses simultaneously, ensuring compatibility with downstream applications such as PCR and NGS, while ensuring robust performance in resource‐limited settings. Ultimately, integrating efficient RNA extraction with sensitive detection in compact, user‐friendly systems represents the most pressing challenge for advancing molecular diagnostics at the POC level.

Isolated viral RNA is instrumental for molecular characterization, enabling the study of virus genetic diversity, evolution, ecology, pathogenesis, mechanism of infection, and epidemiology. Data on mutations and whole genome sequences of circulating viruses in human populations are vital for developing effective control strategies. For instance, genomic RNA of the influenza A/H3N2 virus circulating in Indonesia from 2008 to 2010 was reverse‐transcribed and analyzed for nucleotide sequences of hemagglutinin (HA) and neuraminidase (NA) genes, providing insights into viral genetic dynamics during this period [175]. Viral genetic materials also facilitate the investigation of drug resistance and evolution. Gene sequences of H5N1 avian influenza A, isolated from humans and chickens in Thailand, revealed resistance to amantadine and had multiple basic amino acids at the HA cleavage site, contributing to increased pathogenicity and transmission [187]. Additionally, virus complete genomes can be used to understand host adaptation, virulence, antiviral resistance, and antigenic characteristics, as demonstrated in an Indonesian study on H5N1 avian influenza A isolated from patients between 2008 and 2015 [188]. Another study of 39 H5N1 full genomes from samples collected during poultry influenza outbreaks in 2015–2016 in West Java, Indonesia, showed that genome reassortment occurred in circulating H5N1 viruses [189].

RNA extracted from a novel EBOV strain during the 2017 Likati outbreak in the Democratic Republic of the Congo was utilized to analyze the full EBOV genome sequence, enabling comparisons with other full‐length genomes from previous outbreaks [190]. For genomic characterization of the Indonesian ZIKV, RNA was extracted from culture supernatant and used for complete genome assembly. Phylogenetic analysis revealed that the virus was not closely related to the microcephaly‐associated ZIKV [179].

Isolated viral RNA has been used in genome profiling of SARS‐CoV‐2 circulating in Indonesia and neighboring countries, identifying B.1.466.2 as the predominant variant before the Delta variant outbreak in Indonesia. This analysis suggested virus transmissions between Indonesia and other countries, notably through interactions with Singapore and Japan [191]. A similar approach was applied to characterize the SARS‐CoV‐2 virus responsible for hospital‐acquired infections in a cluster of immunocompromised children in Jakarta [192]. Recently, extracted RNA of SARS‐CoV‐2 was used to identify the distribution of SARS‐CoV‐2 variants and common mutations in Indonesia [32]. Extracted viral RNA is also crucial for the molecular characterization of various other viruses, including DENV [193], CHIKV [194], WNV [48], coxsackievirus [56], measles [79], EV‐71 [166], rhinovirus C [182], human influenza virus A H5N1[195], hepatitis C [196], human rhinovirus Type 14 [197], and O′nyong′nyong viruses [198].

High‐quality viral RNA is a prerequisite for successful genome sequencing and characterization of RNA viruses. This sequence information is critical for designing and developing various vaccines including multiepitope vaccines [199, 200], reverse genetics, recombinant protein subunits, virus‐like particles, viral vectors, DNA, RNA, and antigen‐presenting cells [201–203]. For example, RNA from the CHIKV isolate Ind‐06‐Guj RNA was used for cDNA construction in CHIKV vaccine development [167], and EV‐71 RNA extracted using TRIzol reagent was employed to generate DNA vaccine constructs for developing EV‐71 vaccine candidates [204]. Additionally, a molecular approach involving the extraction of RNA of coxsackievirus B3, cDNA synthesis, and production and purification of recombinant 3C proteinase was used to design specific inhibitors for antiviral drug development [168].

SARS‐CoV (SARS‐CoV‐1) RNA was used to generate cDNA, which served as a template for PCR amplification of the gene encoding the SARS‐CoV nucleocapsid (N) protein. The resulting amplicons were cloned and expressed in *E. coli*, and the recombinant SARS‐CoV N proteins were purified for use as antigens in the development of an ELISA assay for serodiagnosis of SARS [205]. Similarly, RNA from the isolated EBOV strain Gabon 94 RNA was used to create ELISA tests for detecting Ebola infection in humans [206]. Recombinant DENV nonstructural 1 (NS1) proteins were produced from clinical virus isolates for the development of dengue diagnostic tests [169]. In this process, RNA extracted from DENV‐infected cultured cells was used for cDNA synthesis, which then served as a template for PCR amplification of the NS1 gene fragment. The fragment was cloned and expressed in *E*. *coli*, and the recombinant proteins were used to immunize mice, inducing production of NS1‐specific antibodies for DENV diagnostic tests [169]. Additionally, RNA extracted from highly pathogenic avian influenza A H5N1 served as a template for cDNA synthesis, followed by PCR amplification of the full‐length nonstructural protein (NS1) gene. The amplicon was cloned and expressed in *Pichia pastoris* yeast to produce recombinant NS1 proteins, which were then used as antigens for diagnosing highly pathogenic avian influenza infections [170]. A similar strategy was employed using WNV RNA to develop a sensitive and specific ELISA assay for early confirmatory diagnosis of West Nile infection [207].

An automated QIAGEN EZ1 analyzer was used to extract passage‐free SARS‐CoV‐2 genomic RNA using the QIAGEN EZ1 virus mini kit v2.0 for the development of recombinant SARS‐CoV‐2 using reverse genetic approaches. These recombinant viruses are valuable research tools for molecular characterization of SARS‐CoV‐2 [171]. Extraction of Usutu virus genomic RNA using RNeasy plus mini kit (QIAGEN) or miRNeasy mini kit (QIAGEN) was involved in a study investigating the ability of Usutu virus to replicate in the placenta and gain access to the fetus. The Usutu viral RNA was used for RT‐qPCR detection, concluding that the Usutu virus can potentially be transmitted from mother to child [208]. Experimental systems to study the mechanisms underlying the pathogenesis and transmissibility of ZIKV have also been developed. This approach involved the extraction of ZIKV RNA, the construction of a full‐length cDNA clone, and the subsequent generation of recombinant ZIKV [209].

## 7. Conclusion

The ongoing emergence of RNA viruses poses a significant threat to public health, underscoring the need for rapid detection and characterization for implementing specific control measures and limiting viral spread. The extraction of viral RNA is a pivotal step that dictates the success of molecular detection and characterization of RNA viruses. Optimal performance depends not only on the extraction method but also on viral load, sample type, proper handling, and storage conditions. Among the various viral RNA extraction methods, TRIzol‐based, silica column–based, and magnetic bead–based methods remain the most widely used approaches. TRIzol‐based methods quickly denature proteins and stabilize RNA, but involve toxic chemicals and are not easily automated. Silica column–based methods are simple, easy to use, and suitable for large‐scale, high‐throughput processes, though they are relatively expensive. Magnetic bead–based methods are scalable to automation and high‐throughput RNA isolation, but may encounter the risk of cross‐contamination. Recent innovations, such as capture‐based, nuclease‐assisted, and field‐adapted low‐cost methods, offer promising alternatives, though broader validation is needed. To optimize the performance of each viral RNA extraction method, factors such as reagent concentration, reagent availability, RNA stability, cost, and safety need to be considered. Extracted viral RNA has a broad range of applications, including molecular detection, virus characterization, public health countermeasures, diagnostic tests, and research tool development to better understand the biology, evolution, and molecular mechanisms underlying viral pathogenesis and transmission. Integration with downstream processes remains a key challenge: Impurities or inhibitors can compromise PCR sensitivity or hinder NGS library preparation, while sample complexity in matrices such as wastewater or FFPE tissues further complicates workflows. Looking forward, hybrid approaches that merge the reliability of commercial kits with the flexibility and affordability of novel methods are essential. Future research should focus on establishing robust, simple, and cost‐efficient viral RNA extraction methods that provide high‐quality pure RNA suitable for use in POC and resource‐limited settings, as well as scalable and automatable techniques to prepare for large outbreaks and pandemics. Strengthening local capacity for viral RNA extraction is critical for the timely molecular detection and characterization of RNA viruses, ensuring preparedness for future RNA virus epidemics.

## Conflicts of Interest

The authors declare no conflicts of interest.

## Author Contributions

All authors listed have made significant contributions to the development and writing of this article.

## Funding

This research did not receive any specific grant from funding agencies in the public, commercial, or not‐for‐profit sectors.

## Data Availability

Data sharing is not applicable to this article as no datasets were generated or analyzed during the current study.
