# Correction to “Comprehensive Review on Viral RNA Extraction Strategies for Enhanced Molecular Diagnostics”

**DOI:** 10.1155/ipid/9807879

**Published:** 2026-04-20

I. Made Artika, C. N. Ma’roef, E. Johar, F. A. Yudhaputri, and K. S. A. Myint, “Comprehensive Review on Viral RNA Extraction Strategies for Enhanced Molecular Diagnostics,” *Interdisciplinary Perspectives on Infectious Diseases* 2025 (2025): 5579320, https://doi.org/10.1155/ipid/5579320.

The order of Figures 4, 5, 6 and their respective captions in the published article is incorrect. The correct figures and captions are displayed below.

**Figure 4:**
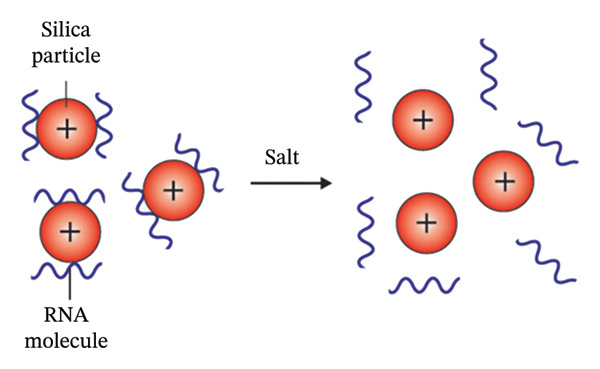
The binding of RNA molecule to silica particles. The affinity between negatively charged RNA molecules and positively charged silica particles causes the selective binding of RNA to silica matrices.

**Figure 5:**
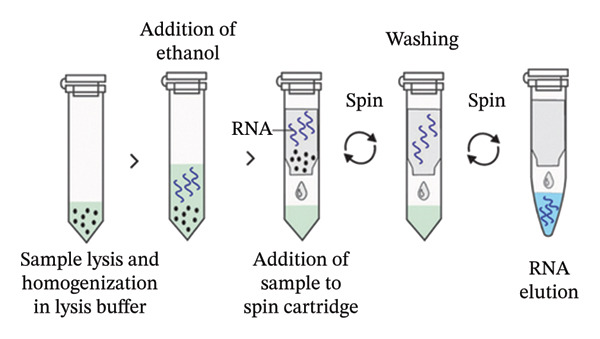
Silica column–based RNA extraction method. The sample is initially lysed and homogenized in lysis buffer containing a chaotropic agent such as guanidinium thiocyanate which releases the RNA. Ethanol is then added to facilitate RNA adsorption to the silica surface. The sample lysate in binding solution is transferred to a spin cartridge, followed by centrifugation. The column is washed by adding a washing buffer followed by centrifugation, and the viral RNA is finally eluted from the column using an elution buffer.

**Figure 6:**
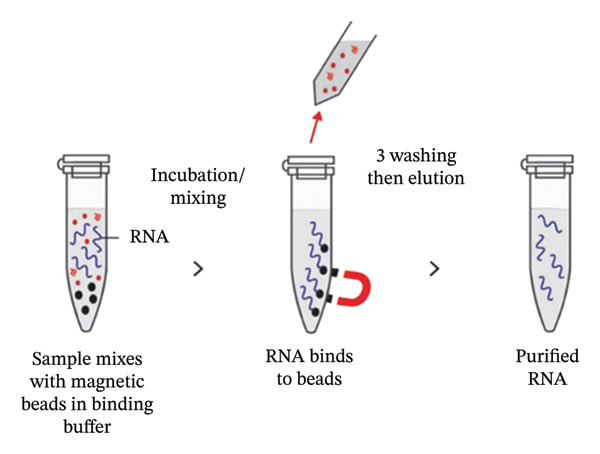
Magnetic beads based RNA extraction method. The lysed sample is incubated with magnetic beads to allow the binding of RNA molecules to the magnetic particles. The viral RNA‐containing magnetic beads are collected by placing them near an external magnetic field. Following removal of supernatant and washing of magnetic beads, the viral RNA is eluted from the magnetic beads using RNase‐free water or an elution buffer.

We apologize for these errors.

